# Posterior cortical atrophy in the age of anti-amyloid treatments: An 11-year retrospective study of eligible patients from the Leenaards Memory Center

**DOI:** 10.1177/13872877251342266

**Published:** 2025-06-25

**Authors:** Paolo Salvioni Chiabotti, Mirco Nasuti, Olivier Rouaud, Gilles Allali

**Affiliations:** 1 Leenaards Memory Center, Department of Clinical Neurosciences, Lausanne University Hospital and University of Lausanne, Lausanne, Switzerland

**Keywords:** Alzheimer's disease, anti-amyloid treatments, Benson's syndrome, donanemab, lecanemab, posterior cortical atrophy

## Abstract

At the era of the anti-amyloid treatments (AAT), it is striking to note posterior cortical atrophy (PCA) cases, the most predictive phenotype of Alzheimer's disease neuropathology, have not been explicitly included or excluded from clinical trials. In a retrospective analysis of the Leenaards Memory Center registry, we identified 41 PCA cases and, applying the recent appropriate use recommendations for lecanemab, estimate high (20%) eligibility rate, that doubles in pure PCA phenotypes with biomarker work-up available (40%). This high proportion of PCA patients eligible for AAT should prompt a timely exploration to assess inclusion/exclusion criteria for these novel therapies.

## Introduction

Posterior cortical atrophy (PCA), also known as Benson's syndrome or visual variant of Alzheimer's disease (AD), is defined as a progressive impairment in visual processing and other posterior cognitive functions, linked with posterior (i.e., parietal, posterior temporal, and occipital) neurodegeneration, with atrophy or hypometabolism at neuroimaging.^1^ No precise estimation on prevalence and incidence of PCA are available, probably due to its rarity and evolving definition.^2^ PCA mostly affects young patients, with a mean age of onset before 60 in the largest available cohorts,^3,4^ and therefore often referred to as a cause of early onset dementia, where it presumably accounts for 7% in a population-based study.^5^ In addition, in an university memory clinic study, it represented around 5% of more than 500 consecutive AD patients.^6^

Intriguingly, even if neuropathological data suggest an extremely high prevalence of AD in PCA patients (94%),^4^ making it the most predictive clinical phenotype of AD neuropathology,^7^ no data on anti-amyloid treatments (AAT) in these patients are available. Moreover, recent AAT randomized clinical trials (i.e., Clarity AD and TRAILBLAZER-ALZ 2) included only early AD patients with amnestic phenotypes.^8,9^

At the era of AAT, where estimations on eligible patients^10^ and economic burden^11,12^ are calculated globally for healthcare planification, assessing the potential eligibility of PCA patients is necessary. We therefore conducted a prevalence study in the Leenaards Memory Center registry to estimate the proportion of PCA patients eligible for AAT.

## Methods

We performed an 11-year retrospective analysis from the Leenaards Memory Center (CLEMENS) registry, from January 2013 to December 2023. The CLEMENS registry gathers clinical, biological and radiological data from patients evaluated at the Leenaards Memory Center, the University Memory Clinic of the Lausanne University Hospital (Switzerland).^13^ We identified all patients where PCA was suspected, either initially or during follow-up. The clinical information of these patients was revised by two independent raters (PSC and OR) and patients meeting the clinic-radiological Crutch criteria for PCA^1^ selected. When disagreement occurred, a clinical case conference involving a third rater (GA) provided a final consensus. They were further classified either as pure PCA phenotype (PCA-pure) or, when fulfilling also core clinical criteria for another neurodegenerative disorder, as PCA-plus.^1^ Subsequently, demographic, biological (CSF) as well as neuroimaging data (including ^18^F-flutemetamol amyloid PET, 18^F^-AV-1451 tau PET, ^18^F-FDG PET, and MRI) were collected. Finally, based on the inclusion and exclusion criteria from lecanemab appropriate use recommendations (AUR),^14^ we estimated the number of PCA patients that could benefit from such treatment.

These AUR include clinical diagnosis of MCI or mild AD dementia, positive amyloid biomarker (PET or CSF), Mini-Mental State Examination (MMSE) between 22 and 30 (or other cognitive screening instrument with a score compatible with early AD), the possibility of being under cognitive enhancing agents (donepezil, rivastigmine, galantamine, or memantine), the absence of radiological (i.e., more than 4 microhemorrhages, a single macrohemorrhage >10 mm at greatest diameter, an area of superficial siderosis, evidence of vasogenic edema, more than 2 lacunar infarcts or stroke involving a major vascular territory, severe subcortical hyperintensities consistent with a Fazekas score of 3, evidence of amyloid beta-related angiitis (ABRA), cerebral amyloid angiopathy-related inflammation (CAA-ri) or other major intracranial pathology that may cause cognitive impairment), clinical (recent (within 12 months) history of stroke or transient ischemic attack, any history of seizures, severe psychosis, major depression, any history of severe immunological, systemic or bleeding disorder – including platelet count < 50,000 or international normalized ratio > 1.5) or pharmacological (patients on anticoagulants) contra-indications.^14^

This analysis was granted by a waiver of the local ethics commission. Figure 2 diagram was made using SankeyMATIC.com.

## Results

**Figure 1.**
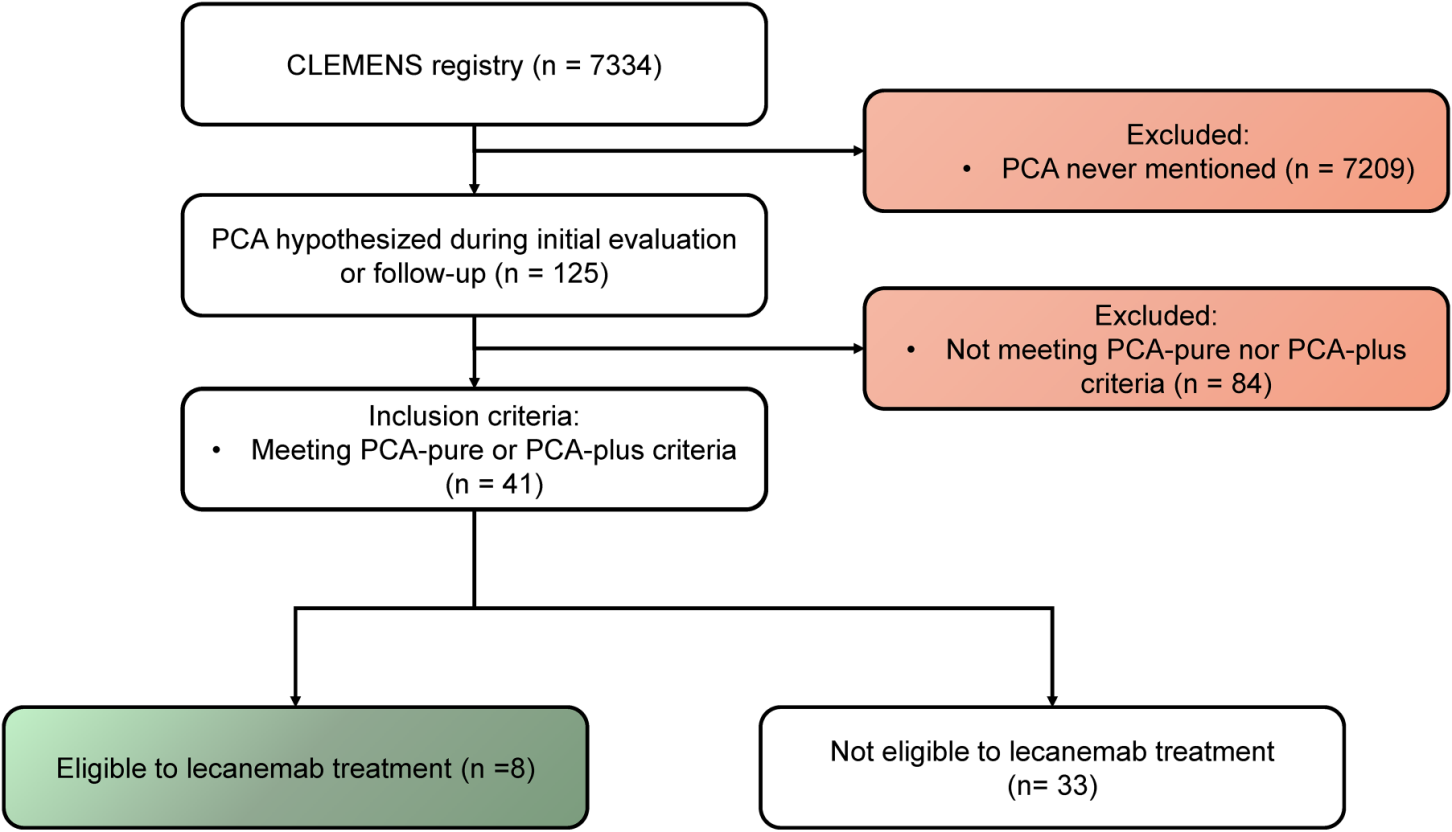
Flowchart illustrating the selection process of the study sample.

From 7334 patients in the CLEMENS registry, we identified 125 patients (1.7%) with suspected PCA. Among them, 41 (30%; 0.5% from the entire registry), meet the Crutch criteria for either PCA-pure (35; 85%) or PCA-plus (6; 15%) syndromes (Figure 1). Mean age at diagnosis was 68 years, women were overrepresented (78%) and mean MMSE at diagnosis was 23. Atrophy in posterior regions at MRI was found in 86% of our cohort, and FDG-PET posterior hypometabolism in the remaining cases (87%), yielding a systematic finding of posterior neuroimaging abnormality. AD was the final diagnosis in 83% of all PCA patients and 94% in PCA-pure cases. Detailed description of their characteristics is summarized in the Supplemental Material (Supplemental Table 1).

Applying the lecanemab AUR inclusion/exclusion criteria,^14^ we were able to identify 8 (20%) eligible patients from PCA cases (Table 1). Mostly female (5; 63%), their mean age at diagnosis was 62 years, younger than non-eligible patients (69 years). Their MMSE score was on average 26, higher than non-eligible (mean MMSE 23) and all but one (7; 88%) had a Clinical Dementia Rating (CDR) score of 0.5. On the other hand, non-eligible patients were almost equally divided in CDR 0.5 (14; 42%) and ≥1.0 (17; 52%). From a phenotypical perspective, all eligible patients were PCA-pure, while in the group of non-eligible patients, PCA-pure was also predominant (27; 82%), with 6 (18%) PCA-plus.

**Table 1.** Clinical characteristics comparing eligible versus non-eligible PCA patients.

	Eligible	Non-eligible	Total
n (%)	8 (20)	33 (80)	41
Mean age at diagnosis – y	62 ± 7	69 ± 8	68 ± 8
Female gender – n (%)	5 (63)	27 (82)	32 (78)
MMSE score - mean	26 ± 2	23 ± 5	23 ± 5
CDR 0.5 – n (%)	7 (88)	14 (42)	25 (61)
CDR >= 1.0 – n (%)	1 (13)	17 (52)	19 (46)
PCA-pure – n (%)	8 (100)	27 (82)	35 (85)
PCA-plus – n (%)	0 (0)	6 (18)	6 (15)

MMSE: Mini-Mental State Examination; CDR: Clinical Dementia Rating; PCA: posterior cortical atrophy.

Interestingly, of the 10 patients meeting the inclusion criteria for lecanemab (35% of PCA-pure), only 2 met exclusion criteria (1 for active psychiatric condition and 1 for cortical siderosis; Supplemental Table 2). On the other hand, 29 PCA-pure cases (82% of PCA-pure) had no exclusion criteria (29; 71%) but 21 of them (72%) were ineligible:10 had low MMSE scores, 7 were missing an amyloid status determination and 4 had a negative amyloid status based solely on CSF Aβ_42_ value. Excluding the 2 PCA-pure cases diagnosed as LBD, we found 24% lecanemab eligibility in the PCA-pure AD cases (n = 33).

In the AD cases, full ATN biomarkers were available in 59%, yielding an A + T + status in 80% of cases. Amyloid status, determined by either CSF or ^18^F-flutemetamol PET was positive in 82% of cases, when performed (65%). Positivity of CSF amyloid status determination was lower (80%) compared to the very rarely performed amyloid PET (100% positivity, in only one case). Furthermore, 4 cases were considered A- based on CSF analysis, performed without the Aβ_42/40_ ratio (not available at the time of the evaluation). Tau status was available in only 59% of cases and was positive by CSF analysis in 90% and by ^18^F-flortaucipir in 100% of cases.

**Figure 2.**
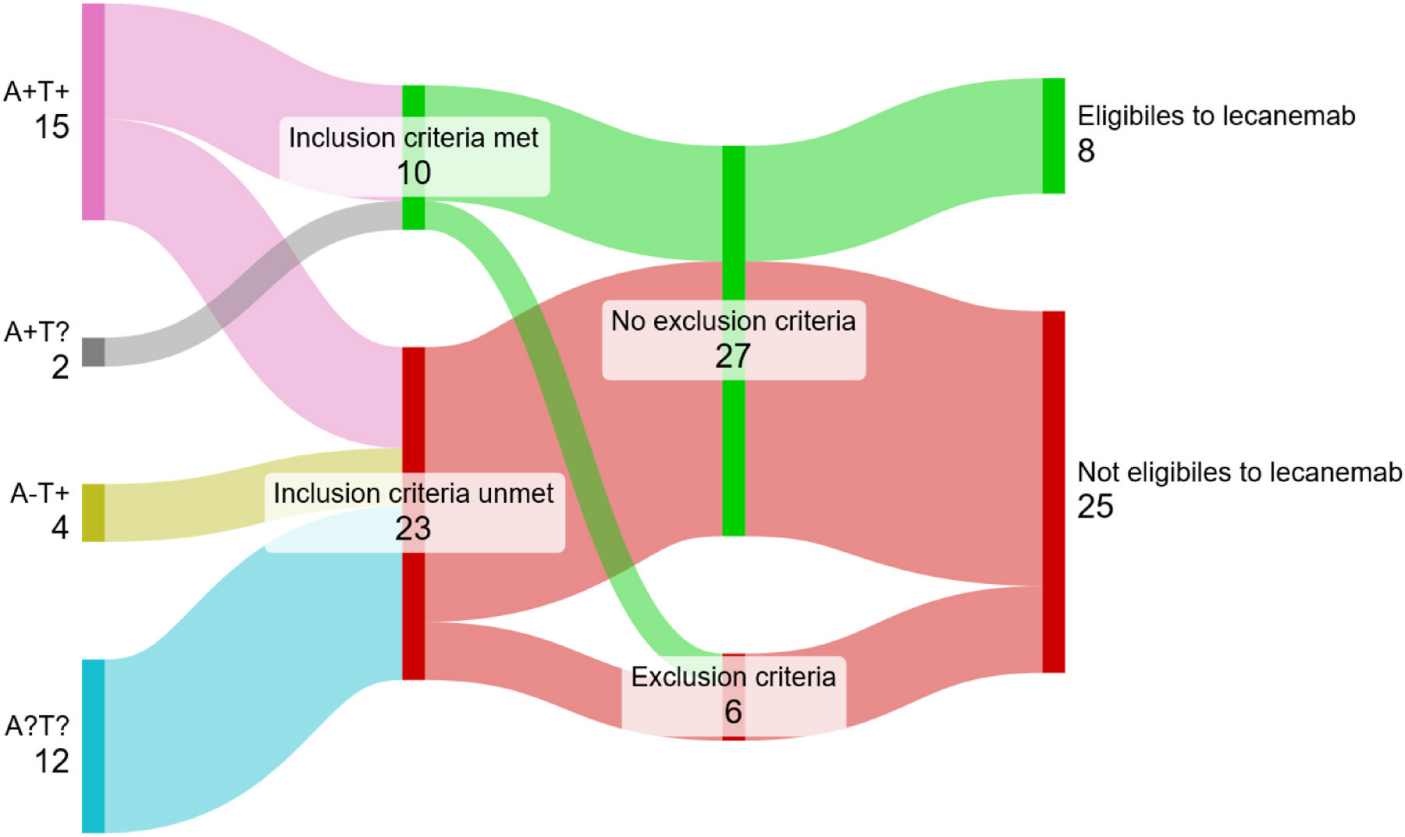
Schematic breakdown representing PCA-pure cases’ lecanemab eligibility, with inclusion and exclusion criteria, stratified according to their biomarker status (n = 33, 2 PCA-pure cases attributed to LBD discarded).

Stratifying eligible patients by their physiopathological biomarker status allowed for a better prediction of lecanemab eligibility (Figure 2). In the A + T + PCA-pure group (n = 15), 8 patients (53%) met the inclusion criteria and only 2 had exclusion criteria (aforementioned), yielding 6 eligible patients (40%, Figure 2, Table 2). Two further eligible patients were identified in the A + T? group, where amyloid status was determined by amyloid PET (Figure 2, Table 2). Finally, while A-T + cases were not considered eligible based on their negative amyloid status (not fulfilling inclusion criteria), none met exclusion criteria, making them potential candidates if their amyloid status was determined by the Aβ_42/40_ ratio analyzed (missing at the time of the clinical assessment) or by an amyloid PET (Figure 2, Table 2).

**Table 2.** Stratification of PCA-pure cases according to their biomarker status (2 PCA-pure cases attributed to LBD discarded).

​	PCA-pure​	A+T+ PCA-pure​	A+T? PCA-pure​	A-T+ PCA-pure	A?T? PCA-pure​
N (%)​	35​	15 (43)​	2 (6)​	5 (14)​	12 (34)​
Mean age at diagnosis – y​	67 +/- 8​	60 +/- 73​	74 +/- 8​	68 +/- 8​	71 +/- 7​
Female gender – n (%)​	26 (74)​	11 (73)​	2 (100)​	4 (80)​	8 (67)​
MMSE score - mean​	23 +/- 5​	22 +/- 4​	29 +/- 5​	26 +/- 5​	23 +/- 6​
CDR 0.5 – n (%)​	18 (51)​	6 (40)​	2 (100)​	3 (60)​	6 (50)​
CDR >= 1.0 – n (%)​	17 (49)​	9 (60)​	0​	2 (40)​	6 (50)​
Fazekas III MRI rating - n (%)​	4 (11)​	1 (7)​	0​	0​	3 (25)​
> 4 microbleeds on MRI – n (%)​	0​	1 (7)​	0​	0​	3 (25)​
Inclusion criteria met – n (%)​	10 (29)​	8 (53)​	2 (100)​	0​	0​
No exclusion criteria met – n (%)​	27 (77)​	12 (80)​	2 (100)​	4 (80)​	9 (75)​
Lecanemab eligibility – n (%)​	8 (23)​	6 (40)​	2 (100)​	0​	0​

## Discussion

This 11-year memory clinic retrospective analysis estimates a high proportion of lecanemab eligibility for PCA patients (20%), higher than in both the cross-sectional Mayo Clinic Study of Aging prevalence study (8%), that likely included only amnestic cases (based on their score on the Wechsler Memory Scale II),^10^ or our previous estimation on AD patients (8%), irrespective of their neuropsychological phenotypes.^15^ It is worth noting that this proportion further raises in PCA-pure cases (23%) and doubles when positive AD biomarkers are available (40%), similar to our previous study on logopenic variant primary progressive aphasia (lvPPA),^16^ another common AD phenotype.^17^

Our biomarker work-up yielded, for amyloid status 82% positivity by CSF analysis and 100% positivity by ^18^F-flutemetamol PET, in line with previous studies.^4^ The proportion of performed amyloid PETs is low because of a national restriction for PET accessibility. Furthermore, CSF Aβ_42/40_ ratio, that clearly improves diagnostic yield,^18^ was not available at the time of the clinical evaluation in 4 cases. On the other hand, tau status was more likely to be positive by^18^F-flortaucipir (100%) than by CSF analysis (90%), as previously reported.^4,19^ Interestingly, in 2 out the 5 cases with both CSF and PET tau status, a discrepancy was observed, with negative CSF p-tau and positive PET, potentially due to the lower CSF p-tau values in PCA, as previously described.^20^

Regarding eligibility, the advanced proportion could even raise with the 4 cases excluded only based on the Aβ_42_ level (i.e., without the Aβ_42/40_ ratio). Applying previously published improvement diagnostic performance of Aβ_42/40_ ratio compared to Aβ_42_ alone,^18^ we would expect at least 2 out of 4 A+ status. Furthermore, from the 9 cases without an ATN status determination otherwise meeting the inclusion criteria, we could speculate, based on our 23% eligibility rate, between 2 and 3 more eligible patients. Thus, if full ATN (with Aβ_42/40_ ratio) was available in all PCA-pure cases, we could reach 34% potential eligible patients.

As already pointed out by some authors,^21^ due to the relative rarity of the disease or the absence of visuo-perceptive items in cognitive scales, data on pharmacological interventions on PCA patients are limited. We suggest rather using functional scales, like the Instrumental Activities of Daily Living scale (IADL) or the Physical Self-Maintenance Scale,^22^ that includes for example dressing abilities, often impaired in PCA, or Clinician's Interview-Based Impression of Change (CIBIC-plus).^23^ On the other hand, biological outcomes, based on clearance of amyloid load on amyloid PET imaging, would act both as proof of target engagement in this peculiar population and as biological monitoring. Applying the centiloid scale^24^ to such patients could add quantitative data. Adding such clinical and biological data in multicentric registries on pharmacological interventions on PCA patients would allow a better appreciation of AAT response in PCA cases.

Finally, it is also important to mention *APOE* status, which by the very nature of this retrospective analysis was not determined. However, we would like to emphasize the under-representation of ε4 genotypes in ACP, whether homozygous or heterozygous,^25,26^ which implies a lower risk of amyloid-related imaging abnormalities. Moreover, since the amyloid load is greater in atypical forms of AD, irrespective of their *APOE* ε4 status,^25,26^ it could be speculated that plaque clearance will be all the more indicated. These two considerations further underline the need to question the response of PCA patients to AAT.

The strengths of our study are both its nature, an unbiased retrospective registry analysis, the high degree of correspondence between our patients and those previously described^4^ and its topicality, being the first epidemiological study that estimates eligible PCA patients to lecanemab treatment. Its limitations are the small number of estimated patients (n = 41) and the lack of modern use of biomarkers available at the beginning of the survey, since diagnostic techniques used in the AD field greatly improved in the last decade.

Taken together, our data suggest a high proportion of potentially eligible PCA-pure patients to lecanemab treatment, a point already suggested by others,^21,27^ especially when an ATN status is determined. These findings should encourage clinicians to promptly obtain an ATN status for patients with a PCA phenotype.

## Supplemental Material

sj-docx-1-alz-10.1177_13872877251342266 - Supplemental material for Posterior cortical atrophy in the age of anti-amyloid treatments: An 11-year retrospective study of eligible patients from the Leenaards Memory Center Supplemental material, sj-docx-1-alz-10.1177_13872877251342266 for Posterior cortical atrophy in the age of anti-amyloid treatments: An 11-year retrospective study of eligible patients from the Leenaards Memory Center by Paolo Salvioni Chiabotti, Mirco Nasuti, Olivier Rouaud and Gilles Allali in Journal of Alzheimer's Disease
