# Supplementary material for: Posterior cortical atrophy in the age of anti-amyloid treatments: An 11-year retrospective study of eligible patients from the Leenaards Memory Center
Source: J Alzheimers Dis. 2025 Jun 25;106(2):512–7. doi: 10.1177/13872877251342266 (PMC12227803; doi:10.1177/13872877251342266)
Supplement: sj-docx-1-alz-10.1177_13872877251342266 - Supplemental material for Posterior cortical atrophy in the age of anti-amyloid treatments: An 11-year retrospective study of eligible patients from the Leenaards Memory Center [file sj-docx-1-alz-10.1177_13872877251342266.docx]

**Supplemental Material**

**Posterior cortical atrophy in the age of anti-amyloid treatments: An 11-year retrospective study of eligible patients from the Lausanne Memory Center**

**Supplemental Table 1.** Demographic, clinical. and biological characteristics of PCA patients

|  | **PCA-pure** | **PCA-plus** | **Total** |
| --- | --- | --- | --- |
| n (%) | 35 (85) | 6 (15) | 41 |
| Mean age at diagnosis – y | 67 ± 8 | 73 ± 11 | 68 ± 8 |
| Female gender – n (%) | 26 (74) | 6 (100) | 32 (78) |
| CDR 0.5 – n (%) | 18 (51) | 3 (50) | 25 (61) |
| CDR >= 1.0 – n (%) | 17 (49) | 1 (17) | 19 (46) |
| MMSE score – mean | 23 ± 5 | 23 ± 5 | 23 ± 5 |
| AD – n (% all PCA; % PCA variant) | 33 (80; 94) | 1 (2; 17) | 34 (83) |
| Positive AD biomarkers – n (% all PCA; % PCA variant; % AD) | 16 (39; 46; 48) | 1 (2; 17; 0) | 17 (41; -; 47) |
| LBD – n (% all PCA; % PCA variant) | 2 (5; 6) | 3 (7; 50) | 5 (12) |
| CBD – n (% all PCA; % PCA variant) | 0 (0; 0) | 2 (5; 18) | 2 (5) |

**Supplemental Table 2.** Detailed inclusion and exclusion criteria analysis in PCA-pure and A+T+ PCA pure cases

|  | | | **pure PCA (%)** | **A+T+ pure PCA (%)** |
| --- | --- | --- | --- | --- |
| Total | | | 35 | 15 |
| Inclusion criteria | | clinical diagnosis of MCI or mild AD dementia | 25 (71) | 9 (60) |
|  |  | positive amyloid PET or CSF | 17 (49) | 15 (100) |
|  |  | physician judgement used for patients outside the 50–90-year age range | 0 | 0 |
|  |  | MMSE 22-30 | 28 (80) | 10 (67) |
|  |  | Extreme BMI (physician's judgement) | 0 | 0 |
|  |  | IAChE but no aducanumab | 2 (6) | 1 (7) |
|  |  | Standard of care for other medical illness | 35 (100) | 15 (100) |
|  |  | Have a care partner | 33 (94) | 14 (93) |
|  |  | Patient and care partner understands potential benefits and harm | n/a | 1 (7) |
| Inclusion criteria met | | | 10 (29) | 8 (53) |
| Exclusion criteria |  | Other cause to cognitive impairment | 2 (6) | 0 |
|  | Neuroradiological contra-indications | > 4 microhemorrhages (< 10 mm) | 0 | 0 |
|  |  | single macrohemorrhage (> 10 mm) | 0 | 0 |
|  |  | era of superficial siderosis | 1 (3) | 1 (7) |
|  |  | evidence of vasogenic edema | 0 | 0 |
|  |  | > 2 lacunar infarcts of strokes | 1 (3) | 0 |
|  |  | Fazekas ≥ 3 | 4 (11) | 1 (7) |
|  |  | evidence of ABRA of CAA-ri | 0 | 0 |
|  |  | major intracranial pathology | 1 (3) | 0 |
|  |  | MRI evidence of non-AD dementia | 0 | 0 |
|  |  | Recent history of stroke, TIA of any history of seizures | 1 (3) | 0 |
|  |  | Mental illness interfering with treatment/cognition | 1 (3) | 1 (7) |
|  |  | Major depression | 0 | 0 |
|  |  | History of immunologic disease or systemic treatment with immunosuppressants | 0 | 0 |
|  |  | Bleeding disorder (Pt < 50000 or INR > 1.5) | 0 | 0 |
|  |  | Anticoagulation drug use | 2 (6) | 0 |
|  |  | Unstable medical condition | 0 | 0 |
| No exclusion criteria met | | | 8 (23) | 3 (20) |
| Eligibility | | | 8 (23) | 6 (40) |
